# Neurogranin and tau in cerebrospinal fluid and plasma of patients with acute ischemic stroke

**DOI:** 10.1186/s12883-017-0945-8

**Published:** 2017-08-30

**Authors:** Ann De Vos, Maria Bjerke, Raf Brouns, Naomi De Roeck, Dirk Jacobs, Lien Van den Abbeele, Kaat Guldolf, Henrik Zetterberg, Kaj Blennow, Sebastiaan Engelborghs, Eugeen Vanmechelen

**Affiliations:** 1 ADx NeuroSciences NV, Technologiepark 4, 9052 Ghent, Belgium; 2 Reference Center for Biological Markers of Dementia (BIODEM), Institute Born-Bunge, University of Antwerp, Antwerp, Belgium; 3 Department of Neurology, Hospital ZorSaam, Terneuzen, The Netherlands; 4 Department of Neurology and Memory Clinic, Hospital Network Antwerp (ZNA) Middelheim and Hoge Beuken, Antwerp, Belgium; 5 Clinical Neurochemistry Laboratory, Sahlgrenska University Hospital, Mölndal, Sweden; 6 Institute of Neuroscience and Physiology, Department of Psychiatry and Neurochemistry, the Sahlgrenska Academy at the University of Gothenburg, Mölndal, Sweden; 7 Department of Molecular Neuroscience, UCL Institute of Neurology, London, UK

**Keywords:** AIS, Neurogranin, Tau, Plasma, CSF, Elisa, Simoa, NIHSS, mRS, Biomarker

## Abstract

**Background:**

While neurogranin has no value as plasma biomarker for Alzheimer’s disease, it may be a potential blood biomarker for traumatic brain injury. This evokes the question whether there are changes in neurogranin levels in blood in other conditions of brain injury, such as acute ischemic stroke (AIS).

**Methods:**

We therefore explored neurogranin in paired cerebrospinal fluid (CSF)/plasma samples of AIS patients (*n* = 50) from a well-described prospective study. In parallel, we investigated another neuronal protein, i.e. tau, which has already been suggested as potential AIS biomarker in CSF and blood. ELISA as well as Single Molecule Array (Simoa) technology were used for the biochemical analyses. Statistical analyses included Shapiro-Wilk testing, Mann-Whitney analyses and Pearson’s correlation analysis.

**Results:**

In contrast to tau, of which high levels in both CSF and plasma were related to stroke characteristics like severity and long-term outcome, plasma neurogranin levels were only correlated with infarct volume. Likewise, CSF neurogranin levels were significantly higher in patients with an infarct volume > 5 mL than in patients with smaller infarct volumes. Finally, neurogranin and tau were significantly correlated in CSF, whereas a weaker relationship was observed in plasma.

**Conclusions:**

These findings indicate that although plasma and CSF neurogranin may reflect the volume of acute cerebral ischemia, this synaptic protein is less likely to be a potential AIS biomarker. Levels of tau correlated with severity and outcome of stroke in both plasma and CSF, in the present study as well as previous reports, confirming the potential of tau as an AIS biomarker.

**Electronic supplementary material:**

The online version of this article (10.1186/s12883-017-0945-8) contains supplementary material, which is available to authorized users.

## Background

Several studies illustrate the potential of neurogranin as biomarker for Alzheimer’s disease (AD), the most common cause of dementia. Concentrations of this post-synaptic protein are higher in CSF of AD patients compared with cognitively healthy individuals [1–7] and such increased levels probably reflect degeneration of synapses, which has been linked to the cognitive decline [8, 9]. Contrary to CSF, neurogranin in plasma does not differ between AD patients and controls, indicating that the protein may not be an AD blood biomarker [3, 10]. Yet, in conditions where sudden severe neurological damage occurs, such as in the event of traumatic brain injury (TBI), increased blood neurogranin concentrations have been found. As recently reported, serum neurogranin levels were elevated in TBI patients compared to controls [11]. This urges the need for additional studies examining the potential value of CSF and plasma neurogranin in conditions associated with brain injury, like acute ischemic stroke (AIS). So far, neurogranin has not been investigated in AIS although this condition is also characterized by sudden brain damage [12].

We therefore studied neurogranin in paired CSF/plasma samples of individuals who have suffered from AIS (*n* = 50). We analyzed CSF and plasma levels on admission to the hospital, as well as plasma concentrations of neurogranin at different time points after stroke onset. For each of these time points, the relationship was explored between neurogranin levels and stroke severity (as defined by the National Institutes of Health Stroke Scale (NIHSS) [13]), as well as long-term stroke outcome (represented by the modified Rankin Scale (mRS) [14]) and infarct volume. To enable a further assessment of neurogranin as biomarker reflecting stroke characteristics, we performed analysis on protein tau as well. Although, to the best of our knowledge, there have been no studies up to date in paired CSF/plasma samples from AIS patients, the brain injury coherent with stroke is seemingly reflected by elevated levels of tau, in both CSF and plasma [15–20]. In the current study, we verified the correlation between tau levels and stroke characteristics.

## Methods

### Study population

The study population is part of the Middelheim’s Interdisciplinary Stroke Study, a project on the clinical, biochemical, neuroimaging, electrophysiological, and neuropsychological evaluation of AIS patients. These individuals were admitted to the stroke unit of Ziekenhuis Netwerk Antwerpen (ZNA)-Middelheim hospital between October 2005 and February 2008. All patients with suspicion of a cerebrovascular accident or transient ischemic attack were assessed for eligibility: patients were included if clinical evaluation and neuroimaging of the brain (within 24 h after admission) were consistent with AIS. A transient ischemic attack was defined as a clinical presentation believed to be secondary to focal cerebral ischemia, but with symptoms lasting less than 1 h and without proof of acute infarction on structural neuroimaging [21, 22]. Biochemical analyses from the Middelheim’s Interdisciplinary Stroke Study project have been reported elsewhere [23–26].

At admission, CSF sampling was performed 8.7 h (6.2 h) (mean (standard deviation)) after onset of stroke symptoms as well as blood collection (5.2 h (5.8 h) after stroke onset). Plasma samples were available at admission and at minimal 3 consecutive time points after stroke onset: at 24 h, 72 h, 7 days, 1 month and/or 3 months post-stroke. CSF samples were obtained by lumbar puncture at the L3/L4 or L4/L5 interspaces, using a 20 Gauge, 3.5 in. Quincke point spinal needle (BD, Belgium), collected in polypropylene cryovials (Nalgene, cat.no.5000–1020 and 5000–0050), immediately frozen in liquid nitrogen and stored at −80 °C until analysis. Venous blood samples were collected in 4.9 mL tubes containing EDTA (Sarstedt Monovette, Germany) and immediately centrifuged at 4000 g during 10 min. Plasma was frozen in liquid nitrogen before storing at −80 °C at the biobank facilities.

The NIHSS was used to assess the neurological deficit by qualified stroke physicians at admission. A NIHSS cut-off score of ≤7 and >8 was used to dichotomize the study population based on stroke severity. To estimate the infarct volume, all patients underwent magnetic resonance imaging (MRI) of the brain at admission or during the first week after stroke onset. The infarct volume was assessed on diffusion weighted MRI by two independent raters as described before [27], using the public domain software Image J (NIH, Bethesda, Md, http://rsb.info.nih.gov/ij). All lesion areas were manually outlined on a slice-by-slice basis and volumes were automatically produced by the multiplication of the total lesion area times the sum of slice thickness and interslice gap. The final infarct volume was calculated as the mean of the infarct volumes obtained by the two raters. The distance between brain infarct and the lateral ventricles was assessed as well. Long-term functional outcome of the AIS was evaluated using the mRS. A favorable outcome was defined by mRS 0 to 3 and a poor outcome as mRS 4 to 6, which is a commonly used and accepted method for dichotomization of the mRS score [28]. From all but one patient, data were available on the CSF/serum albumin ratio, calculated as previously described [29].

### Neurogranin in CSF and plasma

To measure neurogranin in both CSF and plasma, we used a research ELISA described in our previous study [30]. This assay, based on two mouse monoclonal antibodies (mAbs), i.e. ADx403 (clone ADxNGCI2) and ADx451 (clone ADxNGCT1), quantifies specifically neurogranin fragments that are C-terminally truncated at P75. This type of neurogranin is the most abundant form circulating in CSF and plasma based on mass spectrometry data [10]. CSF samples, in duplicate, were analyzed undiluted, whereas plasma samples, also in duplicate, were diluted 1:20 in sample diluent preceding analysis (15 μL). The serial plasma samples were analyzed anonymously, i.e. samples were randomized. Final concentrations of neurogranin in both CSF and plasma were intrapolated (log(X); 5PL) based on a synthetic peptide calibrator. For the analysis of CSF, both the intra-assay (*n* = 2) and inter-plate (*n* = 2) variability of the ELISA was 5% coefficient of variation (CV), based on three run validation (RV) samples with different neurogranin concentrations. For plasma, RV samples with low, mid and high concentrations were quantified, resulting in an intra-assay (*n* = 2) variability of 3%, 2% and 5%CV, and an inter-plate (*n* = 9) precision of 8%, 10% and 14%.

### Total-tau in CSF and plasma

To measure total protein levels of tau in CSF, we used the total-tau ELISA by Euroimmun (Lübeck, Germany). The kit includes lyophilized, ready-to-use calibrators, a standardized protocol, and was performed according to the kit-insert. All CSF samples were run in duplicate. Intra-assay (*n* = 2) variability was 4%CV for RV samples with a low or a high tau concentration. Inter-plate (*n* = 2) precision was 3% and 4%CV respectively.

For quantification of total-tau levels in plasma samples, an in-house research prototype ELISA was used [3]. Undiluted plasma samples were analyzed in duplicate. Tau levels were calculated via intrapolation (log(X); 5PL) based on the calibrator, i.e. *E.coli* recombinant tau 441 (rPeptide, USA), but the quantifications were expressed as arbitrary units (AU) since this concerns a not yet fully developed research assay. The research ELISA enabled detection of tau in 92% of the plasma samples. Two RV samples with different tau levels were run in parallel with the patient samples, resulting in an intra-plate (*n* = 2) variability of 5% and 3%CV, and inter-plate (*n* = 9) precision of 8% and 5%.

Tau plasma levels were also measured using the Simoa assay by Quanterix (Boston, USA), as described elsewhere [31, 32], for which samples, in duplicate, were diluted 4-fold. Based on two samples with different tau concentrations, intra-assay (*n* = 2) variability was 11% and 3%CV, whereas inter-mediate (*n* = 4) precision was 11% and 5%CV. Tau could be quantified in all plasma samples.

As in case of the analysis of neurogranin, tau levels were quantified in randomized plasma samples, blinded from the test performer.

### Statistical analysis

GraphPad Prism 6 was used for statistical analyses and figures. To test normality of data, a Shapiro-Wilk test was performed. In case of a non-normal distribution, all subsequent analyses were based on log-transformed data. To compare quantitative data between two groups, a Mann-Whitney test was applied, whereas correlations were determined using Pearson’s correlation analysis. Results were considered significant for *P*-values <0.05.

## Results

### Study population

Demographic and stroke-related characteristics, as well as stroke syndrome and TOAST (Trial of Org 10,172 in Acute Stroke Treatment) classification are summarized in Table 1. The study population included 23 female and 27 male patients, with mean age at stroke onset 71 ± 14 years. At month 3, 39 patients had a favorable outcome (mRS 0–3) and 11 patients had a poor outcome (mRS 4–6). Of these 11 patients with a poor outcome, 8 died within 3 months after stroke (mRS at month 3 = 6). At month 12, there were 33 patients with a favorable outcome, whereas 10 patients had a poor outcome. Eight of these patients with a poor outcome died within 3 months after stroke, one patient died between month 3 and 12. For the 9 patients who died during follow-up, the cause of death was documented: related to stroke (5 patients), hepatocellular carcinoma (1 patient), post-operative complications of a coronary artery bypass (1 patient) or unknown (2 patients). For 7 patients, the mRS scale was not available at month 12.

**Table 1:** Summary of the demographic, clinical and biomarker data of the AIS patients

Patient characteristics	
Age (years)	71 (±14)
Gender (female/male)	23/27
Time to CSF sampling, at admission (h)	8.7 (± 6.2)
Time to plasma sampling, at admission (h)	5.2 (± 5.8)
Stroke characteristics	
Ischemic stroke/ transient ischemic attack	40/10
NIHSS at admission	6.8 (±7.8)
mRS at 3 months	2.4 (±1.9)
mRS at 12 months	2.1 (±2.3)^a^
Infarct volume (mL)	26.0 (±50.1)^b^
Shortest distance between infarct and ventricles (mm)	5.5 (±8.6)^b^
Stroke syndrome:	
- Lacunar stroke syndrome	8 (±16.0)
- Partial anterior circulation syndrome	20 (±40.0)
- Total anterior circulation stroke	8 (±16.0)
- Posterior circulation syndrome	12 (±24.0)
TOAST classification:	
- Lacunar	8 (±16.0)
- Atherothrombotic	6 (±12.0)
- Cardioemboligenic	17 (±34.0)
- Specific	4 (±8.0)
- Undetermined	13 (±26.0)
- Mimic	2 (±4.0)
Biochemical analysis at admission	
CSF neurogranin (pg/mL)	380 (279–482)
CSF tau (pg/mL)	434 (322–535)

Data are represented as mean (±standard deviation) or median values (interquartiles) as appropriate

^a^ *n* = 43 since for seven patients mRS at 12 m was not available

^b^MRI data was available for a subset of patients: *n* = 33

### CSF neurogranin and tau in relation to stroke severity and outcome

In Table 1, the median CSF concentrations of neurogranin and tau are represented. There was a strong correlation between CSF levels of these two analytes (Pearson’s correlation coefficient *r* = 0.608; *P* < 0.0001).

**Fig. 1:**
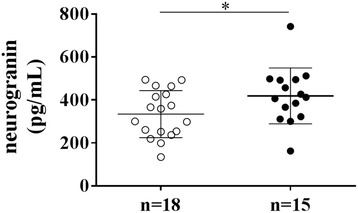
Scatter dot plot of neurogranin in CSF in case infarct volume was dichotomized at 5 mL. The median levels are represented as a line in each dot plot, whereas the bars represent the interquartile range. The open circles represent patient neurogranin levels in case of an infarct volume of 5 mL or smaller (*n* = 18), while the black circles depict a volume greater than 5 mL (*n* = 15). The statistically significant difference between the two groups of stroke patients is represented by * = *P* < 0.05

Furthermore, CSF levels from tau were correlated to stroke severity, represented by the NIHSS values at admission (Table 2). CSF tau was also significantly correlated with mRS score at 12 months after stroke (*r* = 0.403; *P* < 0.01). Notably, there was no correlation between CSF levels of tau and the distance of the infarct to the lateral ventricles, whereas for neurogranin, an inverse relationship was noted (*r* = −0.545; *P* < 0.05). No significant association was seen between the CSF analytes and infarct volume. Yet, in case the infarct volume was dichotomized at 5 mL, CSF neurogranin, but not CSF tau, was significantly increased in the subset of patients with an infarct volume greater than 5 mL (*P* < 0.05) (Fig. 1). Receiver Operating Curve (ROC) analysis demonstrated an Area Under the ROC Curve (AUC)-value of 0.704 (*P* < 0.05).

**Table 2:** Pearson’s correlation analysis of the relationship between CSF neurogranin or CSF tau, and stroke characteristics

Analyte	NIHSS admission	mRS 3 months	mRS 12 months	Infarct volume	Distance to ventricles
CSF neurogranin	−0.075	−0.140	−0.061	0.274	−0.545*
CSF tau	0.356*	0.219	0.403**	0.235	−0.216

The correlation coefficient is presented with the corresponding statistical significance (*P < 0.05; **P < 0.01). Values with statistical significance are in bold and underlined

### Neurogranin and tau in plasma of stroke patients

In this study, all consecutive plasma samples were analyzed in duplicate by ELISA (for neurogranin) or by ELISA and Simoa technology (for tau). A few number of samples were re-analyzed since the %CV between duplicates was above 20%, i.e. *n* = 5 for neurogranin, *n* = 7 for tau by ELISA and *n* = 5 for tau by Simoa. As consecutive plasma samples from patients were always run on the same plate, the entire patient’s series was re-analyzed in that case. Since plasma tau levels obtained by ELISA correlated significantly with levels quantified by the Simoa assay (*r* = 0.678; *P* < 0.0001) (see Additional file 1), only tau levels resulting from ELISA are discussed in the following paragraphs, unless stated otherwise.

**Fig. 2:**
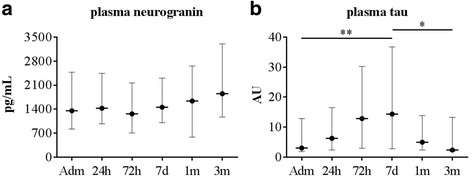
Median plasma levels of (**a**) neurogranin and (**b**) tau at all time points. The bars depict the interquartiles. Levels of tau are represented as arbitrary units, since quantified by a research ELISA. Statistical significance, based on Tukey’s multiple comparison analyses on log-transformed data, is represented by * = *P* < 0.05, ** = *P* < 0.01

Plasma levels of neurogranin and tau are depicted as median values with interquartile range in Fig. 2. There was no strong relationship between neurogranin and tau in plasma. Levels of both analytes only correlated weakly (*r* = 0.313; *P* < 0.05) 7 days after stroke onset.

At none of the time points, CSF and plasma levels of neurogranin were correlated. CSF tau levels were related to plasma tau levels at admission (*r* = 0.353; *P* < 0.05) and 1 m after stroke onset (*r* = 0.357; *P* < 0.05).

Plasma neurogranin did not correlate with stroke severity, as represented by NIHSS, nor with long-term outcome, i.e. with mRS at 3 months or 12 months after stroke, at any of the time points (Table 3). A relationship was observed between infarct volume, measured at admission, and concentrations of plasma neurogranin at 24 h (*r* = 0.510; *P* < 0.01) and 72 h (*r* = 0.478; *P* < 0.01) after stroke onset. Notably, plasma levels of neurogranin 72 h after stroke were weakly inversely related to the CSF/serum ratio of albumin (*r* = −0.330; *P* < 0.05) and, at 3 months after stroke onset, to the distance between the infarction and the ventricles (*r* = −0.688; *P* < 0.05). Stronger correlations with stroke characteristics were seen for tau. Plasma levels of tau were significantly related to stroke severity, stroke outcome as well as infarct volume at several time points after stroke (Table 3).

**Table 3:** Pearson’s correlation analysis of the relationship between plasma neurogranin or tau and stroke characteristics

Plasma neurogranin	NIHSS admission	mRS 3 m	mRS 12 m	Infarct volume	Distance to ventricles	CSF/serum albumin ratio
Admission	−0.073	−0.214	−0.214	0.288	−0.212	−0.186
24 h	−0.037	0.067	0.024	0.510**	−0.132	−0.163
72 h	0.135	0.114	−0.038	0.478**	−0.115	−0.330*
7d	−0.110	−0.144	−0.192	0.330	−0.249	−0.266
1 m	−0.003	−0.207	−0.149	0.353	−0.154	−0.218
3 m	0.201	0.078	0.203	0.231	−0.688*	−0.007
Admission	0.230	0.201	0.143	0.380*	−0.503*	0.236
24 h	0.379*	0.286	0.233	0.528**	−0.492*	0.197
72 h	0.451**	0.434**	0.411**	0.614***	−0.302	0.202
7d	0.484***	0.459**	0.465**	0.753****	−0.408	0.063
1 m	0.120	0.314	0.295	0.392	−0.138	0.202
3 m	−0.239	0.203	−0.050	0.243	−0.474	0.021

The correlation coefficient is presented with the corresponding statistical significance (*P < 0.05; **P < 0.01; ***P < 0.001; ****P < 0.0001). The values with statistical significance are in bold and underlined

To further explore the value of plasma tau in reflecting stroke characteristics, we calculated the AUC-values for the analyte at each time point, wherefore stroke severity or outcome were dichotomized. As illustrated in Table 4, strong predictive power was demonstrated at time points between 24 h and 1 m after stroke onset. When assessing the AUC-values based on tau quantified by the Simoa assay, which allowed quantification of tau-levels in an additional 22 samples, statistical significance increased at time points (see Additional file 2).

**Table 4:** Value of tau as plasma AIS biomarker reflecting stroke severity and long-term outcome

		AUC value	*P*-value
NIHSS	At admission	0.581	0.355
	24 h	**0.701**	**0.022**
	72 h	**0.751**	**0.004**
	7d	**0.730**	**0.009**
	1 m	0.672	0.113
	3 m	0.518	0.890
mRS 12 m	At admission	0.638	0.198
	24 h	0.645	0.180
	72 h	**0.750**	**0.019**
	7d	**0.804**	**0.005**
	1 m	**0.850**	**0.027**
	3 m	0.783	0.348

Severity (NIHSS) or outcome at 12 months (mRS 12 m) were first dichotomized: patients with NIHSS scores ≤7 were pooled versus NIHSS >7, or with mRS scores 0–3 versus scores 4–6. ROC analysis was performed at all time points on both groups for tau, quantified by ELISA. Statistically significant AUC-values are in bold font and underlined

## Discussion

Although progressive brain damage, as in case of AD, is clearly not reflected in plasma levels of neurogranin, it remains to be investigated how acute brain injury affects the blood levels. In the current study, we focused on acute ischemic stroke and observed a relationship between increased plasma neurogranin and infarct volume. Yet, neither stroke severity nor long-term outcome were reflected by neurogranin in plasma, or CSF. Levels of axonal protein tau, on the other hand, were significantly associated with stroke severity and long-term outcome, indicating that different neuronal proteins may entail different kinds of information, at least in AIS.

Due to the high plasma concentrations of neurogranin in normal individuals, i.e. more than 1000 pg/mL, small increases are possibly not detected in plasma in case of slow progressive neurodegeneration like AD [3, 10]. Only in conditions of acute severe brain trauma, such as TBI, neurogranin may serve as potential plasma biomarker. Serum neurogranin concentrations in TBI cases were recently reported as significantly higher than in controls [11]. In our study on AIS, we monitored plasma neurogranin from admission to the hospital until 3 months post-stroke. This aspect was suggested in the recent study [11] on neurogranin in TBI since this could offer insights in the temporal profile of the protein in blood, following acute brain damage. We observed a trend in increasing plasma levels of the protein, with the highest concentration at 3 m. Blood brain barrier dysfunction has been reported in stroke [33], which could allow progressive leakage of brain-derived neurogranin into the plasma. However, this contradicts the observation of the lowest plasma neurogranin levels at 72 h being inversely related to a high CSF/serum albumin ratio, suggesting that lower neurogranin levels may be linked to a dysfunctional blood brain barrier. To the best of our knowledge, this is the first report on neurogranin in AIS and further studies are needed, preferentially in moderate or even severe conditions of brain damage. Based on the relatively low NIHSS scores, the patients suffered from a mild stroke in the current study, which is corroborated by the rather low CSF tau values at admission. CSF tau has been reported to substantially increase in case of stroke, i.e. about a 4-fold increase in case of higher NIHSS values [15]. Hence, more severe stroke may result in higher neurogranin levels. Nonetheless, our current dataset suggests that neurogranin may not have great value as plasma biomarker, or as CSF biomarker, for acute brain injury in case of stroke. Besides only observing a trend in increasing levels in function of time, plasma levels were inversely related to the distance between the infarct and the ventricles, which represents a confounding factor. In case the infarct occurs at a larger distance from the ventricles, this could lead to a possible underestimate of the infarct volume. Moreover, even though levels in plasma and CSF of neurogranin were significantly associated with infarct volume, levels were not related to stroke severity, neither to long-term outcome, which are clinical relevant characteristics. Perhaps neurogranin could still have value as biomarker, reflecting infarct volume, when combined with other proteins, such as GFAP, and UCH-L1, which show potential in case of TBI [34, 35]. Or, it could even be combined with tau since our findings are strongly indicative for a high performance of tau, even as single analyte, as measure for stroke severity and outcome in AIS.

Plasma tau significantly peaked 7 days after stroke, where levels were linked to stroke severity, i.e. NIHSS, long-term outcome, represented by the mRS, as well as infarct volume, which confirms prior reports [19, 20]. No relationship with the distance between the infarct and the ventricles, nor with the CSF/serum albumin ratio was seen at that time point. Similarly, CSF levels of tau, which correlated to the plasma levels of the protein, were related to NIHSS assessment at admission and mRS rating 12 months after stroke. This result was in line with previously published studies on tau in CSF from AIS patients [15, 17, 36]. Interestingly, CSF tau at admission was not associated with infarct volume unlike plasma tau. Since plasma tau only peaked 7 days after stroke, it is plausible that CSF tau does not adequately reflects infarct volume at admission, but rather 72 h to 1 week after stroke onset. This was actually suggested by Hesse and colleagues, who demonstrated increasing CSF tau levels over time that peaked one or 3 weeks after stroke onset. Only at maximum CSF tau concentrations, there was a correlation with infarct volume [16, 17]. This corroborates earlier findings on higher CSF tau levels in patients with large infarcts in the subacute stage, i.e. less than 1 month after stroke onset, compared to patients in the acute stage, i.e. less than 1 week [37]. Importantly, the latter report mentioned that even small infarct volumes resulted in elevated CSF tau levels several days after stroke.

Although plasma tau was the best candidate biomarker in the current study, compared to neurogranin, the search continues for non-invasive AIS biomarkers providing an early evaluation of the stroke. Ideally, physicians can gauge the severity and long-term outcome of the acute brain damage on admission to the hospital. Most likely this will be enabled by analysis of a panel of proteins, possibly including plasma tau.

## Conclusion

Based on the present findings, we conclude that neuronal proteins like neurogranin and tau do not entail the same information in brain injury concurring with acute ischemic stroke. Neurogranin may be a plasma biomarker for acute ischemic stroke, unlike for AD, by reflecting infarct volume. However, plasma tau has added clinical relevant value compared to neurogranin, since plasma levels of this analyte reflect stroke severity and long-term outcome. Also in CSF, tau is the most promising candidate biomarker for stroke.

## Additional files

Additional file 1: Correlation.tiff. Correlation analysis of plasma tau levels measured with the ELISA (*n* = 241) or Simoa (*n* = 263) assay. The Pearson’s correlation coefficient, on log-transformed data, was 0.678 (*P* < 0.0001). (TIFF 53 kb)

Additional file 2: Plasma AIS biomarker tau.xls. Value of tau as plasma AIS biomarker reflecting stroke severity and long-term outcome. Severity (NIHSS) or outcome at 12 months (mRS 12 m) were first dichotomized: patients with NIHSS scores ≤7 were pooled versus NIHSS >7, or with mRS scores 0–3 versus scores 4–6. ROC analysis was performed at all time points on both groups for tau, quantified by Simoa. Statistical significant AUC-values are depicted in bold font and underlined. (XLSX 11 kb)

## Abbreviations

AD: Alzheimer’s disease
AIS: Acute ischemic stroke
AU: Arbitrary units
AUC: Area under the curve
CSF: Cerebrospinal fluid
CV: Coefficient of variation
ELISA: Enzyme linked immunoSorbent assay
MRI: Magnetic resonance imaging
mRS: Modified rankin scale
NFL: Neurofilament light
NIHSS: National institutes of health stroke scale
ROC: Receiver operating characteristic
RV: Run validation
Simoa: Single molecule array
TBI: Traumatic brain injury
TOAST: Trial of Org 10,172 in acute stroke treatment
ZNA: Ziekenhuis Netwerk Antwerpen
