# Supplementary figures and images for: Neurogranin and tau in cerebrospinal fluid and plasma of patients with acute ischemic stroke
Source: BMC Neurol. 2017 Aug 30;17:170. doi: 10.1186/s12883-017-0945-8 (PMC5577791; doi:10.1186/s12883-017-0945-8)

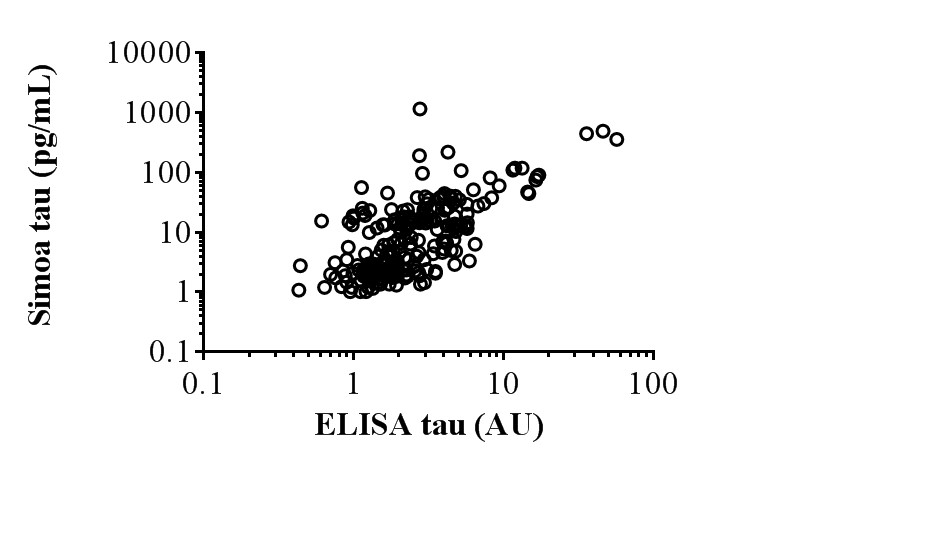

Supplement: Supplementary file 1 — Correlation.tiff. Correlation analysis of plasma tau levels measured with the ELISA (n = 241) or Simoa (n = 263) assay. The Pearson’s correlation coefficient, on log-transformed data, was 0.678 (P < 0.0001). (TIFF 53 kb) [file 12883_2017_945_MOESM1_ESM.tif]
